# Editorial: Decoding the genetics of milk fat traits

**DOI:** 10.3389/fgene.2022.1059219

**Published:** 2022-11-09

**Authors:** Xiang Cao, Dongxing Sun, Zhi Chen

**Affiliations:** ^1^ College of Animal Science and Technology, Yangzhou University, Yangzhou, China; ^2^ Shandong Shengli Bioengineering Co., Ltd., Shandong, China; ^3^ Joint International Research Laboratory of Agriculture & Agri-Product Safety, Ministry of Education, Yangzhou University, Yangzhou, China

**Keywords:** milk fat, GWAS, circRNA, dairy breeding, fatty acid

It is undeniable that milk and dairy products provide the maximum nutritional value in ancient and modern times when nutrients are scarce. As animal husbandry has developed, a considerable increase in the proportion of livestock meat and dairy products in people’s dietary structure has been brought about. Even so, it is still possible to increase the per capita intake of fresh milk and dairy products in many countries and regions. As important sources of essential fatty acids and various unsaturated fatty acids, bovine and sheep milk contain a series of bioactive fatty acids, such as conjugated linoleic acid (CLA), docosahexaenoic acid (C22:6 n-3, DHA), and arachidonic acid (C20:4n-6, AA). Milk fat is not only a major factor that affects flavor and nutritional value, but also considered to be a significant economic trait in dairy breeding.

A crucial factor affecting the quality of fresh milk is the regulation of mammary fatty acid metabolism, which involves the expression, network regulation and signal transduction of multiple genes (including circular RNAs). Several factors affect milk fat percentage, including genetics, feeding management and nutrition. Additionally, the expression of enzymes and transcription factors is extremely important for milk fat synthesis. In a network of competitive endogenous RNAs (ceRNAs), long-stranded non-coding RNAs (LncRNAs) and circular RNAs (circRNAs) act on milk fat-related genes through indirect interactions with microRNAs (miRNAs) or RNAs, thereby triggering positive transcriptional effects.

CircRNAs, a type of non-coding RNAs (ncRNAs), perform important molecular functions in mammary gland development and lactation. As revealed by RNA sequencing (RNA-Seq), a high level of circ-015343 expression is observed in sheep mammary tissue during peak lactation. Moreover, circ-015343 suppresses the expression levels of marker genes involved in milk fat synthesis, including Fatty Acid Binding Protein 4 (FABP4), Acetyl-CoA Carboxylase Alpha (ACACA) and Sterol Regulatory Element-Binding Protein 1 (SREBP1) (Wu et al.). Additionally, according to a circRNA-miRNA-mRNA interaction network, it is also known to bind some miRNAs to regulate the expression of lactation-related functional genes during mammary gland development (Lu et al.).

Non-lactating breast tissue showed the highest levels of MiR-199a-3p regulation compared with peak lactation. In sheep mammary epithelial cells (OMECs), MiR-199a-3p inhibits milk lipid synthesis by inducing low triglyceride levels and reducing expression of lipoprotein lipase (LPL), ACACA, heart-type fatty acid binding protein (FABP3), stearoyl-coenzyme A desaturase (SCD) and fatty acid synthase gene (FASN) (Wang et al.). Besides, milk lipid synthesis is also affected by short/branched chain acyl-coenzyme A dehydrogenase (ACADB). This is because knockdown of the ACADB gene in daily cows results in the reduction of cholesterol (CHOL), triglycerides (TGs) and free fatty acids (FFA) levels in mammary epithelial cells (BMECs). Furthermore, there has also been a change in the expression levels of *ACADL*, *ACOX2, ACAT2* and *FABP3*, which are functional genes involved in lipid metabolism (Jiang et al.). A random regression test-day model and genome-wide association analysis (GWAS) are used to estimate and analyze genetic parameters of milk yield (MY), milk fat percentage (MFP), milk fat amount (MFY), milk protein percentage (MPP) and milk protein amount (MPY). Results provide insights into the basis of quantitative genetics and the genetic structure of milk-related traits in dairy cows (Lu et al.).

As international dairy cattle and dairy goat breeding have developed, the concept of balanced breeding has gradually diversified the breeding objectives that are no longer limited to milk production traits. There is an increasing focus on functional traits, such as udder health, reproduction and disease resistance, which are being incorporated into breeding programs with extensive and long-term technical requirements. As a result of nanotechnology, dairy animal diseases can be treated in therapeutics, diagnostics, tissue engineering, vaccine production, animal welfare and other fields. A number of benefits can be achieved by using nanomaterials in cattle and sheep feeds, including the inhibition of harmful pathogens, regulation of rumen fermentation processes, and an increase in immunity and reproductive performance (Wang et al.). There are numerous complex processes participating in and cooperating during the early stages of mammalian embryonic development. As the sheep embryo develops in its maternal environment, many epigenetic factors regulate its gene expression, resulting in spatial and temporal effects. DNA methylation is an epigenetic mechanism that plays a key role in silencing gene expression and maintaining genomic stability. During sheep pre-implantation embryonic development, demethylation occurs from the 8- to 16-cell stage and methylation occurs from the 16- to 32-cell stage (Zhang et al.). Notably, C4BPA, a type of multifunctional genes, not only regulates lipid metabolism in BMECs through the PPAR signaling pathway, but also participates in the inflammatory response through the TLR-4/NF-κB, and complement-coagulation crosstalk pathways. These genes can be used as target genes to select and breed cows with high mastitis resistance and high milk fat production (Iqbal et al.).

This Research Topic focuses on the genetics of milk fat traits in dairy cows, dairy goats, and other dairy animals, and the identification of genetic and functional genes involved. Additionally, the development of more accurate genomic prediction methods for daily animals is also underway, which is a prerequisite for successful genetic improvement and breeding. An in-depth analysis of the regulatory mechanism of mammary fatty acid metabolism can serve an experimental basis for improving milk quality and a reference for improving the dietary fat intake. Considering the long-term perspective, in addition to the genetic level, it is also important to note that the selection and breeding of dairy animals will require better quality semen, more comfortable breeding environment, and the conservation and utilization of animal diversity.

